# Supplementary material for: Deepening Inside the Pictorial Layers of Etruscan Sarcophagus of Hasti Afunei: An Innovative Micro-Sampling Technique for Raman/SERS Analyses
Source: Molecules. 2019 Sep 19;24(18):3403. doi: 10.3390/molecules24183403 (PMC6766820; doi:10.3390/molecules24183403)
Supplement: Supplementary file 1 [file molecules-24-03403-s001.pdf]

Deepening inside the pictorial layers of Etruscan sarcophagus of Hasti Afunei: an innovative micro-sampling technique for Raman/SERS analyses

Rossella Gagliano Candela[a], Livia Lombardi[b], Alessandro Ciccola[b], Ilaria Serafini[b], Armandodoriano Bianco[b], Paolo Postorino[c], Lorella Pellegrino[d], Maurizio Bruno[a]*

a Department STEBICEF, University of Palermo, Viale delle Scienze Ed. 17, 90128, Palermo, Italia;
b Università degli Studi di Roma "La Sapienza", Dipartimento di Chimica, Piazzale Aldo Moro 5, 00185 - Rome, Italy
cUniversità degli Studi di Roma "La Sapienza", Dipartimento di Fisica, Piazzale Aldo Moro 5, 00185 - Rome, Italy
d Regional Center for the Design and Restoration of Cultural Heritage, Regional Department of Cultural Heritage and Sicilian Identity, Via dell'Arsenale 52, 90142, Palermo, Italia

*corresponding author: maurizio.bruno@unipa.it

The Hasti Afunei sarcophagus is a large Etruscan urn, made up of two chalky alabaster monoliths. Dated from the last quarter of the third century BC, it was found in 1826 in the small town of Chiusi (Tuscany- Il Colle place) by a landowner, Pietro Bonci Casuccini, who made it part of his private collection. The noble owner's collection was sold in 1865 to the Royal Museum of Palermo (today under the name of Antonino Salinas Regional Archaeological Museum), where it is still displayed. The sarcophagus is characterized by a complex iconography that is meticulously illustrated through an excellent sculptural technique, despite having subjected to anthropic degradation and numerous restorative actions during the last century. During the restoration campaign carried out between 2016 and 2017, a targeted diagnostic campaign was carried out to identify the constituent materials of the artefact, the pigments employed and the executive technique, in order to get an overall picture of conservation status and conservative criticalities. In particular, this last intervention has allowed the use of the innovative micro-sampling technique, patented by the Cultural Heritage research group of Sapienza, in order to identify the employee of lake pigments through SERS analyses. Together with this analysis, Raman and NMR technique have completed the information requested by restorers, for what concerns the wax employed as protective layers, and allowed to rebuild the conservation history of the sarcophagus. In fact, together with the identification of red ocher and yellow ocher, carbon black, Egyptian blue and madder lake, pigments compatible with the historical period of the work, modern pigments (probably green Paris, chrome orange, barium yellow, blue phtalocyanine) have been recognized, attributable with not documented intervention during the eighteenth and twentieth centuries.

SUPPLEMENTARY MATERIALS

NMR analysis – Identification of surface protective

Experimental

NMR spectra were performed on 3 superficial sampling of the sarcophagus to determine the composition of the organic material. The samples were suspended in 600 µL of $CDCl_3$. One-dimensional ^{1}H spectra were obtained through 128 scans for each sample on a Bruker Avance III 400 spectrometer (Bruker Spectrospin, Karlsruhe, Germany) under the following conditions: 9.4 T at 298K, acquisition of the given 64K points FID, with a spectral window of 15ppm, 128 scans and an intersection time of 10.28s, for a total time of 15s between consecutive scans.

Discussion

For spectrum 1 (fig.1S), referable to sample 1, we observe the presence of saturated fatty acids and paraffins (characteristic signals $(CH_2)^n$ area is in the region between δ: 1.22-1.37 ppm and that of CH_3 in the region included between δ: 0.85-0.95 ppm). The paraffins resonate with chemical shifts compatible with the presence of oxidation on the chain (and the signal at δ: 3.60 ppm is indicative of this phenomenon). It is not possible to see signals attributable to glycerols or unsaturations (the absence of unsaturation is indicative of the absence of oleic acid and other unsaturated ones). Furthermore, in this sample, it is possible that the signals of saturated fatty acids (in the region between δ: 2.25-2.35 ppm) and those referable to hydroxylated paraffins are also compatible with the presence of beeswax.

As for spectrum 2 (Fig.2S), compounds similar to those found in spectrum 1 are observed, in addition it is possible to observe signals attributable to polar heads, in particular the phosphatidylcholine (δ: 3.29, t; 3.46, s; 3.62, t). The systems related to paraffin are even more deshielded than those observed in samples 1 and 3. The absence of signals originating from CH_2 attached to carbonyls, in this sample unlike samples 1 and 3, suggests that the origin of paraffin either from oil distillation or similar processes (comparison in Fig.9).

With regard to spectrum 3 (Fig. 3S) bands similar to sample 1 are observed but at different concentrations.

In Figure 4S, a comparison between the spectrum of the sample 2 and of the sample 3 is shown. In the area between 2.50 - 6 ppm, it is possible to observe more accentuated oxidation phenomena in the sample 2 than in sample 3. This information could suggest that two or more protectives have been used; it is not possible to establish certainly if in mixture or in two different steps.

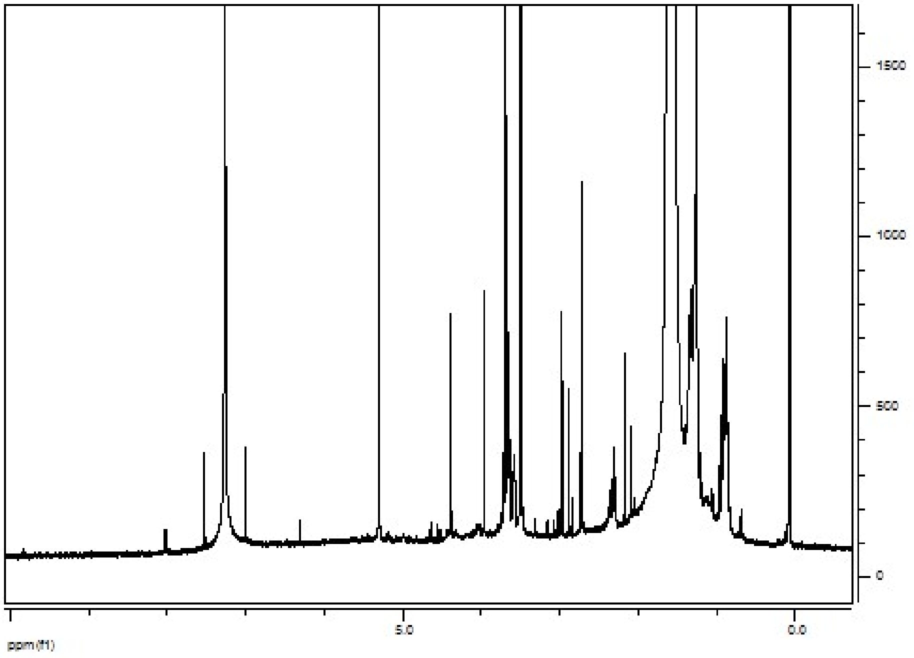

Fig. 1S - ^{1}H NMR ($CDCl_3$, 400 MHz) of sample 1.

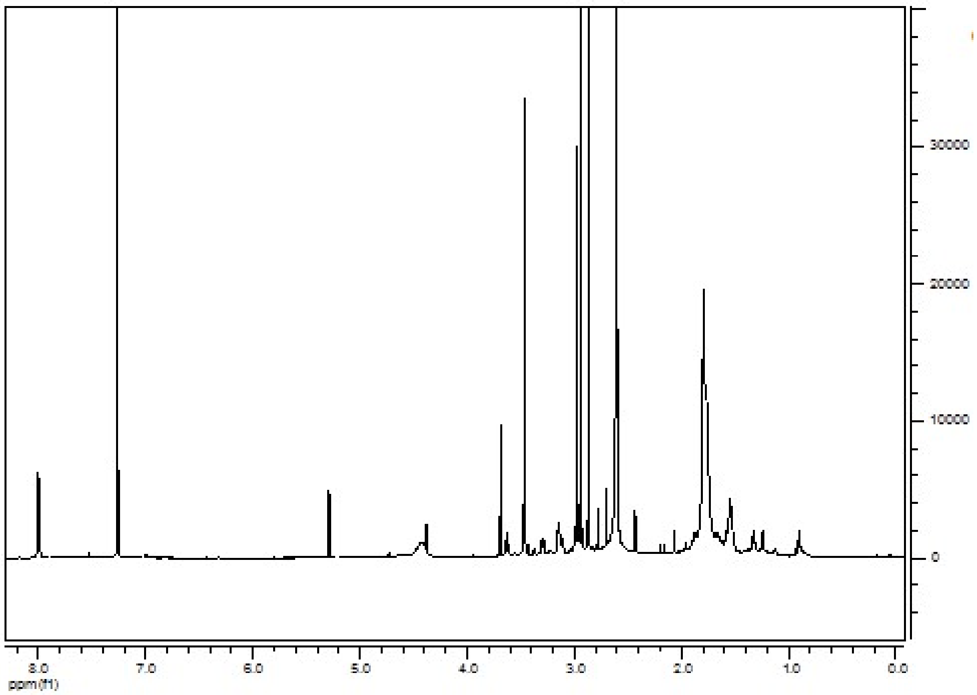

Fig. 2S - ^{1}H NMR ($CDCl_3$, 400 MHz) of sample 2.

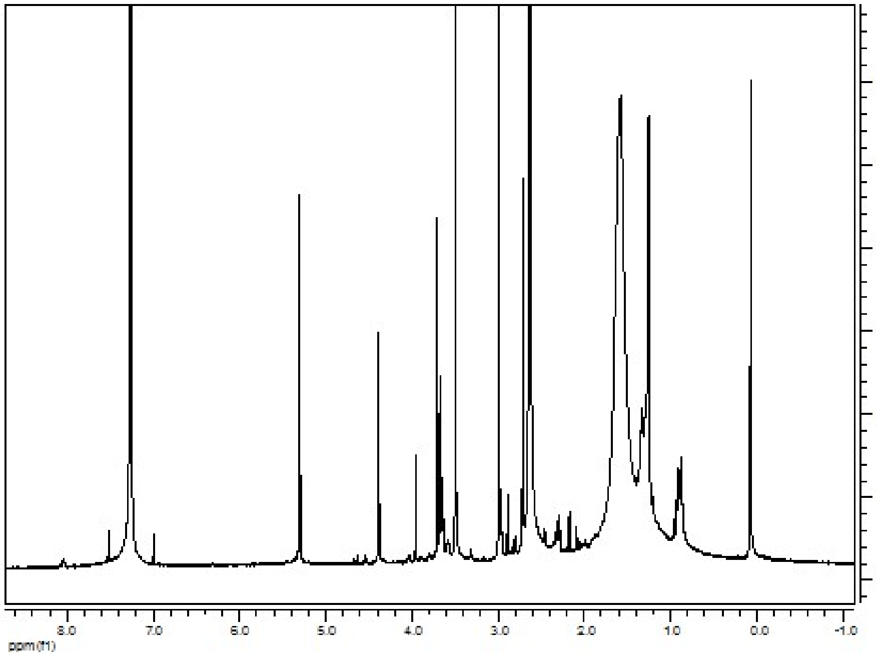

Fig. 3S - ^{1}H NMR ($CDCl_3$, 400 MHz) of sample 3.

Sample 2

Sample 3

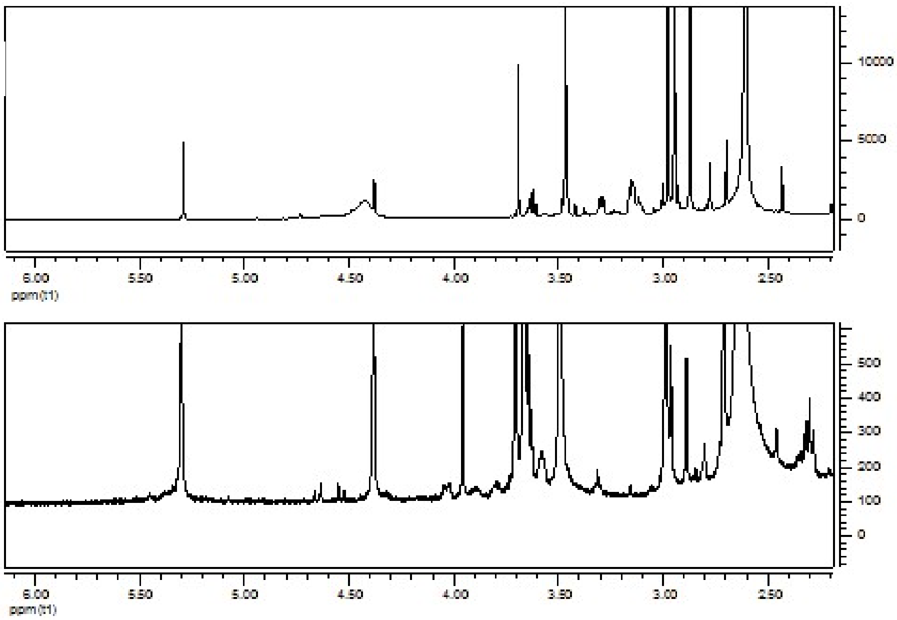

Fig. 4S - Comparison between sample 2 and sample 3, in the area between 2.50 – 6 ppm.
